# A mixed-methods exploration of social networks, self-efficacy, and life satisfaction in rural Chinese high schools

**DOI:** 10.3389/fpsyg.2025.1501328

**Published:** 2025-07-17

**Authors:** Ping Zhu, Tingting Wang

**Affiliations:** ^1^Graduate School of Education, Daejin University, Pocheon-si, Gyeonggi-do, Republic of Korea; ^2^Weifang University of Science and Technology, Weifang, Shandong, China

**Keywords:** social capital, academic self-efficacy, life satisfaction, social networks, rural students, narrative inquiry, family support

## Abstract

**Introduction:**

This mixed-methods study explored how social networks influence academic self-efficacy and life satisfaction among rural Chinese high school students.

**Methods:**

The study involved 454 students in the quantitative phase, utilizing the Social Network Index (SNI), Academic Self-Efficacy Scale (ASES), and Satisfaction with Life Scale (SWLS). The qualitative phase included interviews with 28 students.

**Results:**

Quantitative results revealed that network centrality (*r* = 0.34, *p* < 0.001) and density (*r* = 0.28, *p* < 0.001) positively correlated with academic self-efficacy. Network centrality (*r* = 0.20, *p* < 0.001) and network size (*r* = 0.22, *p* < 0.001) were positively correlated with life satisfaction. Females exhibited higher academic self-efficacy than males [*t*_(452)_ = 3.52, *p* < 0.001], and network density increased with grade level [*F*_(2, 451)_ = 6.78, *p* = 0.001]. Qualitative themes highlighted supportive friendships as empowering, competitive pressures within dense networks as both motivating and stressful, and broader networks (including family) as vital for well-being.

**Discussion:**

These findings emphasize the dual role of social networks in fostering academic confidence and life satisfaction while also introducing competitive stressors. This is particularly relevant in rural contexts where family support is crucial. Implications include the need to promote supportive peer relationships and mitigate the negative effects of competition.

## 1 Introduction

Social networks and social capital are crucial in shaping educational outcomes by providing access to essential resources, support, and information for academic success (Coleman, 1988; Putnam, 2000; Dika and Singh, 2002). In this study, social capital is defined as the networks, norms, and trust that enable cooperation and mutual benefit, with a focus on how these elements influence rural Chinese high school students' academic and emotional outcomes. In educational settings, these networks include relationships with peers, family, and educators, all of which influence students' motivation, self-efficacy, and overall wellbeing (Eccles and Roeser, 2015; Epstein, 2019). The dynamics of social networks are especially important in rural areas, where limited resources and socio-economic challenges can worsen educational inequities (Mishra et al., 2023; Roscigno et al., 2006).

In rural China, educational disparities arise from socio-economic and resource-based inequalities that restrict students' access to quality education and support systems (Talbert-Johnson, 2004; Owings and Kaplan, 2024). Students from lower socio-economic backgrounds often lack strong social capital, including mentorship and academic resources, which negatively affects their academic self-efficacy and life satisfaction (Gorski, 2017; Shifrer et al., 2013). Understanding the intersection of social capital and educational inequities is essential for examining how social networks influence academic outcomes in these contexts (Bernardi and Ballarino, 2016; Baquedano-López et al., 2013).

Existing research has thoroughly explored the role of social capital in education, showing that strong and interconnected social ties improve academic performance by providing emotional support and access to resources (Putnam, 2000; Dika and Singh, 2002). Bonding social capital within close-knit groups, such as family and close friends, offers essential support for managing academic tasks, fostering a sense of belonging and motivation (Ahn, 2012; Murray et al., 2020). Additionally, bridging and linking social capital connect students to diverse networks and institutional resources, further boosting their academic self-efficacy (Lin, 1999; Kyne and Aldrich, 2020).

However, social networks present both opportunities and challenges. While supportive peer relationships aid learning and provide emotional support, they may also generate stress through competition and social comparison (Fox and Moreland, 2015). Social media use and exposure to idealized portrayals of peers' success can foster inadequacy and lower motivation (Feinstein et al., 2013; Vogel et al., 2015). Understanding these complex impacts requires considering individual characteristics and peer group dynamics (Juvonen et al., 2019; Samad et al., 2019). Family support, distinct from peer influence, offers crucial emotional stability and long-term guidance, impacting academic self-efficacy and life satisfaction (Harris and Robinson, 2016; Roksa and Kinsley, 2019). In rural settings with limited institutional resources, family involvement becomes even more critical for academic competence and persistence (Baquedano-López et al., 2013), contributing to better performance, resilience, and future aspirations (Fan and Williams, 2010; Llorca et al., 2017).

Despite the established importance of social networks and family support, limited research exists on their combined impact on academic self-efficacy and life satisfaction among rural Chinese high school students. Most studies focus on urban settings or specific academic areas, leaving a gap in understanding rural educational challenges (Mishra et al., 2023; Roscigno et al., 2006). Furthermore, the dual role of social networks in providing both support and pressure in these contexts warrants more attention (Festinger, 1954; Fox and Moreland, 2015). This study addresses these gaps by exploring how social networks shape academic self-efficacy and life satisfaction among rural Chinese high school students. Employing a mixed-methods approach, it combines quantitative social network analysis (SNA) with qualitative narrative inquiry to offer a comprehensive view of peer relationships, family support, and social capital's influence on students' academic and emotional wellbeing. The study operationalizes social capital through network structures (measured via Social Network Analysis) and relational qualities (explored through interviews), linking these to academic and emotional outcomes in rural settings. By focusing on rural settings, the study aims to examine the socio-economic and resource-based disparities that impact students' educational experiences in these areas (Talbert-Johnson, 2004; Owings and Kaplan, 2024).

This research significantly contributes to the literature on social networks, particularly in rural China, by exploring the complexities of peer relationships and the dual effects of support and pressure on academic outcomes. It highlights the crucial role of family support in resource-limited rural areas and examines educational inequities through the lens of social capital, demonstrating how access to networks, resources, and support systems contributes to academic disparities. By addressing these interconnected factors, the study offers valuable insights for educators, policymakers, and stakeholders aiming to improve educational outcomes and promote equity in rural settings. The findings can inform targeted interventions to build social capital, strengthen support networks, and mitigate the negative effects of competition and social comparison.

## 2 Literature review

### 2.1 The foundation of social capital and networks in academic success

Social capital refers to the networks, norms, and trust facilitating cooperation and mutual benefit within society (Bhandari and Yasunobu, 2009; Demir, 2021; Portes, 1998; Putnam, 2000). In educational contexts, social capital profoundly shapes academic outcomes by providing essential resources, emotional support, and guidance through interpersonal networks, significantly influencing students' motivation, academic self-efficacy, and life satisfaction (Eccles and Roeser, 2015; Epstein, 2019; Roksa and Kinsley, 2019). These two constructs, academic self-efficacy and life satisfaction, were selected as key dependent variables in this study because they represent critical dimensions of students' academic and overall wellbeing, both of which are theorized to be significantly influenced by social capital and network dynamics.

Social capital manifests in three distinct forms, each uniquely supporting academic achievement and wellbeing. Bonding capital, derived from close relationships within homogeneous groups like family and friends (Ahn, 2012; Murray et al., 2020), offers critical emotional and academic support for navigating educational challenges. It facilitates access to resources and mentorship (Kutash et al., 2013), fosters belonging that enhances academic motivation (Uslu and Gizir, 2017), and strengthens self-efficacy—the belief in one's ability to succeed academically (Bandura, 1997). This emotional backing from bonding capital directly bolsters self-efficacy by reinforcing confidence in academic capabilities, a key driver of persistence and performance (Pajares, 1996; Zimmerman, 2000). Furthermore, the sense of security and support provided by strong bonding ties contributes to higher levels of life satisfaction (Baumeister and Leary, 1995). In contrast, bridging capital connects individuals across diverse groups (Claridge, 2018; Kyne and Aldrich, 2020), providing access to novel information, diverse perspectives, and broader opportunities. Such connections stimulate intellectual growth, improve critical thinking, and develop social skills through collaborative interactions (Dufur et al., 2013; Jensen and Jetten, 2015). Bridging capital's exposure to varied resources and role models can enhance self-efficacy by offering new strategies for success, while also boosting life satisfaction through a sense of connection across social boundaries and increased opportunities (Lin et al., 2001). Linking capital ties students to institutions and authority figures (Dika and Singh, 2002; Dufur et al., 2013), granting access to institutional support such as scholarships and counseling, essential for disadvantaged students overcoming systemic barriers (Mishra, 2020; Woolcock, 1998). This institutional support from linking capital can improve self-efficacy by providing tangible resources for academic achievement and elevate life satisfaction by reducing stress from educational inequities and enhancing future prospects.

These forms of social capital underpin social networks that significantly influence academic support and motivation. Peer networks, as primary sources of bonding capital, create collaborative learning environments that enhance academic self-efficacy and engagement by encouraging mutual responsibility (Topping, 2005; Van der Zanden et al., 2018). Such peer interactions surpass mere emotional support, facilitating structured opportunities for mutual accountability and collective achievement (Wang et al., 2018). Peer collaboration provides mastery experiences and vicarious learning—key sources of self-efficacy (Bandura, 1997)—while fostering belonging that supports life satisfaction by fulfilling needs for relatedness and companionship (Ryan and Deci, 2000). Similarly, family networks boost motivation by reinforcing long-term educational aspirations. Families establish stable, supportive environments, promoting intrinsic motivation and sustained academic effort through consistent expectations and encouragement (Fan and Chen, 2001; Hill, 2015; Ma et al., 2016; Riley, 2016). Unlike immediate peer-driven dynamics, family support emphasizes perseverance toward future-oriented goals. This consistent support from family directly enhances self-efficacy by instilling confidence and contributes to life satisfaction by providing a secure and predictable environment. Educators, representing linking capital, substantially contribute to student motivation through constructive feedback and mentorship. Teachers who emphasize effort over innate ability encourage students to view challenges as growth opportunities, thus promoting self-efficacy and improvement (Dweck, 2006; Ricard and Pelletier, 2016; Yeager and Dweck, 2020). Educator-led mentorship programs further clarify the long-term benefits of persistence, aligning current academic efforts with future career aspirations (Crisp and Cruz, 2009; Hernandez et al., 2017). Teacher feedback serves as verbal persuasion, a critical self-efficacy source (Bandura, 1997), while mentorship enhances life satisfaction by linking academic effort to personal purpose and future success.

Across these networks, fostering a sense of belonging is crucial for student wellbeing and engagement. Inclusive, supportive academic environments reduce isolation, sustaining motivation and participation (Booker, 2007; Juvonen et al., 2019; Van Herpen et al., 2020). This sense of belonging, facilitated by strong social connections, directly contributes to higher life satisfaction by fulfilling the fundamental human need for relatedness (Baumeister and Leary, 1995). Emotional support, alongside instructional feedback, builds resilience against academic challenges (Korpershoek et al., 2020), further bolstering self-efficacy. In higher education, mentorship, community ties, and digital platforms—such as online groups—support underrepresented students by enhancing collaboration and belonging, essential for motivation in complex settings (Nández and Borrego, 2013; Tiryakioglu and Erzurum, 2011; Tzur et al., 2023).

Social capital positively impacts academic outcomes (Jensen and Jetten, 2015). Strong family and community networks boost performance through shared expectations (Coleman, 1988), while home- and school-based social capital enhance aspirations and achievement (Dika and Singh, 2002; Huang, 2009; Parcel et al., 2010). Human and social capital interactions further improve competencies (Daly et al., 2021). Teacher networks, like professional learning communities, create supportive environments, improving outcomes (Demir, 2021; Jensen and Jetten, 2015; Mishra, 2020). However, excessive bonding capital can limit opportunities and reinforce inequalities (Lin, 1999; Portes, 1998), and sustaining social capital is challenging in resource-scarce areas (Mishra, 2020; Moshtari and Vanpoucke, 2021).

In summary, social capital shapes academic success through networks providing support and resources. Inclusive networks, leveraging peer, family, and teacher dynamics, improve outcomes, particularly for disadvantaged students, by enhancing self-efficacy and life satisfaction (Baumeister and Leary, 1995; Ryan and Deci, 2000). Therefore, understanding the interplay between social capital, social networks, academic self-efficacy, and life satisfaction is crucial for comprehending the holistic wellbeing and academic trajectories of students, especially in the context of rural Chinese high schools.

### 2.2 The multifaceted influences of social connections on student wellbeing

Social networks within academic settings exert dual influences on students, providing both essential support and potential pressure that shape their academic outcomes and overall wellbeing, including life satisfaction—the global evaluation of one's quality of life (Diener et al., 1985; Nández and Borrego, 2013; Samad et al., 2019). These networks can foster collaboration, emotional support, and motivation, but may also introduce stress, competition, and distractions detrimental to academic focus (Fox and Moreland, 2015). Thus, a balanced understanding of social connections' advantages and disadvantages is necessary to elucidate their impact on life satisfaction and self-efficacy.

Supportive social networks facilitate academic assistance and peer collaboration, creating shared learning experiences that promote accountability, engagement, and motivation (Kilday and Ryan, 2022; Wang and Eccles, 2013). Peer relationships often extend beyond academics, offering emotional support that buffers against stress and enhances students' capacity to manage school pressures (Lee et al., 2023). Strong peer ties foster a sense of belonging, positively correlating with academic engagement and success (Wang et al., 2018). Information shared among peers also shapes students' academic self-concept and motivation, further influencing performance (Dudovitz et al., 2017; Rizzuto et al., 2009). These peer dynamics enhance self-efficacy through collaborative mastery experiences and boost life satisfaction by meeting needs for relatedness and support, per self-determination theory (Ryan and Deci, 2000).

However, social networks can also create pressure, particularly in competitive academic environments. According to Festinger's (1954) social comparison theory, evaluating oneself against others' achievements—often idealized on social media—can heighten stress, envy, lower self-esteem, and reduce motivation Feinstein et al., 2013; Vogel et al., 2015. Excessive social media engagement may distract from academic tasks, contributing to cognitive overload and academic fatigue (Junco, 2015). Additionally, pressure to maintain an idealized online presence and concerns about privacy can further detract from academic focus (Bright et al., 2015). Peer relationships may foster unhealthy competition; while peer accountability can motivate students, competition within networks may also generate significant stress. Frequent social media use during study periods has been linked to poorer academic outcomes, undermining peer support's benefits in high-pressure settings (Felisoni and Godoi, 2018; Giunchiglia et al., 2018). Such competitive pressures can erode self-efficacy by fostering doubt and diminish life satisfaction through increased anxiety and isolation.

Distinct from peer and educator networks, family support significantly influences students' academic and emotional wellbeing (Harris and Robinson, 2016; Roksa and Kinsley, 2019). Family relationships provide unconditional emotional backing and long-term guidance, critical to fostering academic resilience and success. Family support notably enhances academic self-efficacy, as parents create environments that instill confidence in students' abilities and encourage perseverance despite immediate setbacks (Bandura, 1994; Fan and Williams, 2010; Llorca et al., 2017; Schunk and Zimmerman, 2007; Yap and Baharudin, 2016). Moreover, stable and supportive family environments promote positive self-image and emotional stability, key components of life satisfaction. Family support acts as a buffer, reinforcing self-efficacy through consistent encouragement and enhancing life satisfaction by providing a secure emotional base.

Broader family networks, including siblings and extended relatives, contribute emotional and academic resources. Siblings provide peer-like support, while extended family offer guidance complementing formal education (Galindo and Sheldon, 2012; Gueldner et al., 2020). These networks enhance academic performance and life satisfaction, mitigating socio-economic disadvantages (Baquedano-López et al., 2013; Becker and Luthar, 2002). Consistent family involvement improves GPA over time, bridging achievement gaps, and fostering long-term aspirations, especially for disadvantaged students (Cheng et al., 2012; Ishimaru et al., 2016).

Family support is particularly significant for ethnic minority students in STEM, emphasizing educational value and aiding complex academic paths (Hardy et al., 2018; Niehaus and Adelson, 2014). It also prevents dropout and sustains engagement by promoting accountability and vision (Gil et al., 2021; Kim, 2014). Synergy between family and school environments further boosts self-efficacy and academic engagement, linking family background to academic outcomes (Stubbs and Maynard, 2017; Weiser and Riggio, 2010).

In summary, social networks in academia offer support but also pose pressures, requiring balance to maximize benefits. Family support, providing emotional stability and guidance, significantly enhances resilience, self-efficacy, and life satisfaction, making these constructs key outcomes for studying network effects.

### 2.3 Social capital as a lens for understanding educational inequities

Educational inequities disproportionately affect marginalized students, significantly hindering academic achievement and closely relating to disparities in social capital (Gorski, 2017; Shifrer et al., 2013). Social capital, defined as access to networks, resources, and supportive relationships, either mitigates or exacerbates these inequities. Students with strong social capital have greater educational opportunities, while those lacking it—often from lower socio-economic backgrounds—face substantial barriers (Coleman, 1988). Unequal resource distribution, intensified by socio-economic and geographical factors, deepens these inequities, negatively impacting students' academic self-efficacy and life satisfaction (Talbert-Johnson, 2004). Limited social capital undermines self-efficacy by restricting access to supportive networks and diminishes life satisfaction by increasing stress and isolation.

Racial disparities in resource access and social capital further compound educational inequities (Roscigno and Ainsworth-Darnell, 1999). Minority students frequently lack mentors and role models essential to fostering academic self-efficacy and success (Kumar, 2023). Limited social experiences and inadequate institutional support widen socio-economic achievement gaps (Thiem and Dasgupta, 2022). Such disparities are especially pronounced in rural communities, where underdeveloped educational infrastructure leads to fragmented social capital and lower postsecondary attainment (Mishra et al., 2023; Roscigno et al., 2006). Addressing these inequities requires strengthening social capital through mentorship, community engagement, and peer support. In rural contexts like China, where this study is situated, these gaps directly affect self-efficacy and life satisfaction, making them critical lenses for analysis.

Deep-seated socio-economic inequalities within educational systems further perpetuate disparities, benefiting affluent students with established networks while leaving disadvantaged students behind (Tranter, 2012). Targeted initiatives, including school-community partnerships, mentorship, and parental involvement programs, are crucial for enhancing disadvantaged students' social capital, thus improving their academic performance, belonging, self-efficacy, and motivation (Bernardi and Ballarino, 2016).

Educational inequities have significant psychological impacts, as marginalized students face increased stress and reduced belonging due to limited social capital (Quintana and Mahgoub, 2016). Interventions like mentorship and peer support can provide emotional backing, while equitable resource distribution ensures access to both material resources and resilience (Jones et al., 2020). Chronic underfunding in schools serving low-income and minority students restricts quality education and social capital development (Cole-Henderson, 2014), perpetuating achievement disparities (Darling-Hammond, 2013). Equitable funding policies, including investments in extracurriculars and teacher training, are essential for equal opportunities (Owings and Kaplan, 2024).

Opportunity gaps in mathematics and science further deepen inequities, with minority students often lacking advanced coursework and qualified teachers, hindering development and network-building (Gorski, 2017). Reforms should expand access, improve training, and foster mentorship, but policies like school choice must be carefully designed to avoid increasing segregation (Darling-Hammond, 2015). Addressing inequities requires tackling socio-economic, geographical, and psychological factors linked to social capital disparities. Policies should balance equitable resources with mentorship and peer support to promote equity, enabling success for all students. This underscores self-efficacy and life satisfaction as key outcomes reflecting the toll of these disparities.

### 2.4 The present study

This study investigates academic self-efficacy and life satisfaction as key indicators of how social networks, embedded within the broader framework of social capital, influence the academic success and overall wellbeing of rural Chinese high school students. These constructs were selected as dependent variables because they directly reflect the dual impact of social capital: its role in facilitating academic achievement through enhanced confidence and motivation (self-efficacy) and its contribution to emotional wellbeing and overall quality of life (life satisfaction). Academic self-efficacy, defined as students' belief in their ability to succeed in specific academic tasks such as studying for exams or completing assignments (Bandura, 1997), is critical because it drives motivation, effort, and persistence, leading to better grades, test scores, and engagement in learning (Honicke and Broadbent, 2016; Multon et al., 1991; Pajares, 1996; Richardson et al., 2012; Zimmerman, 2000). Students with high self-efficacy set challenging goals and persevere through difficulties, making it a vital measure for understanding academic outcomes in rural settings where resources may be limited. Life satisfaction, reflecting students' overall evaluation of their quality of life based on their experiences and relationships (Diener et al., 1985), captures emotional health, which is crucial during adolescence—a period of significant developmental changes (Erikson, 1968; Proctor et al., 2009). High life satisfaction is linked to better mental health, stronger social relationships, and improved academic adjustment, making it essential for assessing wellbeing in rural Chinese high schools where socio-cultural challenges may impact students' lives (Huebner et al., 2000; Seligman, 2002).

Social networks play a significant role in shaping academic self-efficacy. Supportive peers, family, and educators offer feedback, collaborative learning opportunities, and encouragement that build students' confidence in their academic abilities (Bandura, 1997; Epstein, 2019). For example, peer study groups provide mastery experiences, observing successful peers offers vicarious learning, and positive feedback from teachers serves as verbal persuasion—all key sources of self-efficacy (Eccles and Roeser, 2015). Similarly, social networks influence life satisfaction by meeting fundamental needs for connection and support. Positive relationships reduce loneliness, foster a sense of purpose, and enhance wellbeing, while negative interactions, such as competitive pressures amplified by social comparisons, can lower satisfaction (Vogel et al., 2015). In rural China, where institutional resources may be scarce, family, and peer networks are particularly critical for both academic confidence and emotional health.

We analyzed gender differences in academic self-efficacy and life satisfaction based on research suggesting potential variations (Ma et al., 2016; Llorca et al., 2017) and social role theory (Eagly and Wood, 2016), considering the influence of traditional gender roles in rural China. Grade-level differences were explored using adolescent development theory (Erikson, 1968) and social network research (Wang et al., 2018), anticipating network evolution to impact self-efficacy and life satisfaction.

Based on the literature, this study tests the following hypotheses:

1. Stronger social networks (higher centrality and density) will be associated with higher academic self-efficacy (Coleman, 1988; Lin, 1999).
2. Supportive social networks (larger size and higher density) will be positively associated with life satisfaction, while networks with high competition will be negatively associated with life satisfaction (Baumeister and Leary, 1995; Feinstein et al., 2013; Vogel et al., 2015).
3. Family support will moderate the relationships between social networks and both academic self-efficacy and life satisfaction, strengthening positive effects in resource-limited rural areas (Fan and Williams, 2010; Llorca et al., 2017).
4. Female students will report higher academic self-efficacy than male students (Ma et al., 2016; Eagly and Wood, 2016).
5. Network density will increase with grade level, and students in higher grades will report higher academic self-efficacy and life satisfaction (Erikson, 1968; Wang et al., 2018).

Additionally, we explored the role of other demographic variables, such as age, in relation to self-efficacy and life satisfaction, without specific hypotheses due to limited prior research in this context. These hypotheses align with the study's goal of examining how social networks, as social capital, influence rural students' academic and emotional lives, aiming to understand how network structures and relational support help or hinder students in rural Chinese high schools to inform educational improvements and wellbeing in these underserved areas.

## 3 Methods and materials

### 3.1 Subjects

This study employed a two-phase mixed-methods design to examine rural high school students in mainland China. Participants were recruited using purposive sampling from two rural high schools located in Sichuan Province (Southwest China) and Heilongjiang Province (Northeast China). These provinces were selected to capture variation in rural contexts, such as economic activities and cultural influences, while focusing on high school students. School principals and teachers assisted with participant recruitment, ensuring representation from all grade levels (10–12).

A total of 454 students participated in the quantitative phase (39% male, *n* = 177; 61% female, *n* = 277; average age 17.2 years, *SD* = 0.8). The gender imbalance reflected the higher number of female students in the sampled schools and potentially greater female participation willingness. This was addressed by including gender as a control variable in analyses. Ethical guidelines were followed, with informed consent obtained from all participants and parental consent for those under 18. Data was collected anonymously.

Ethical guidelines were strictly followed throughout the study. Informed consent was obtained from school principals, teachers, and each student participant. Parental consent was secured for students under 18. Students received an information sheet explaining the study's purpose, procedures, and their right to withdraw at any time. All questionnaires were completed anonymously, and identifying information was removed during data analysis. The study protocol received approval from the Institutional Review Board at Daejin University.

For the qualitative phase, a purposive sub-sample of 28 students was selected from the initial SNA sample to ensure diversity in network characteristics and academic self-efficacy scores, aiming to explore the relationship between social capital and academic self-efficacy. Selection criteria included varying levels of academic self-efficacy (low: < 45, medium: 45–60, high: >60), network size (small: 0–2 friends, medium: 3–5 friends, large: ≥6 friends), and network density (low: < 0.2, medium: 0.2–0.4, high: >0.4). Participants were chosen to represent a mix of these categories, accounting for grade level and aiming to reflect the quantitative sample's gender distribution (11 males, 39%; 17 females, 61%; average age 17.3 years, *SD* = 0.7). This slightly deviated from the intended gender balance to prioritize diversity across key variables, as detailed in Table 1. The same ethical procedures were followed, with written informed consent obtained prior to semi-structured interviews conducted in private school settings and audio-recorded.

**Table 1:** Distribution of qualitative phase participants by key characteristics.

**Characteristic**	**Category**	**Number of participants**
Gender	Male	11
	Female	17
Grade	10	9
	11	10
	12	9
Academic self-efficacy	Low (< 45)	7
	Medium (45–60)	14
	High (>60)	7
Network size	Small (0–2)	8
	Medium (3–5)	12
	Large (≥6)	8
Network density	Low (< 0.2)	9
	Medium (0.2–0.4)	10
	High (>0.4)	9

### 3.2 Instruments

This study employed a combination of instruments to collect data on social networks, academic self-efficacy, and life satisfaction among rural high school students in China.

#### 3.2.1 Social network analysis (SNA)

The Social Network Index (SNI) developed by Cohen et al. (1997) was adapted for this study. While the core name-generator question remained unchanged, asking students to list their closest friends within the school, certain modifications were implemented to enhance its suitability for the rural Chinese high-school context. Students were instructed to “list up to six classmates you talk with at least three times a week and whom you would feel comfortable asking for academic help.” Pilot work showed that six nominations balanced recall accuracy with questionnaire length; fewer than 5% of respondents wished to list more names. This design aligns with social capital theory by targeting bonding social capital—close-knit ties that provide academic and emotional support (Ahn, 2012)—and allows measurement of network structures central to the construct (Putnam, 2000).

First, the original instructions were simplified and translated into Chinese, ensuring clarity for the target population. Second, the pilot test revealed that some students were hesitant to list friends due to concerns about confidentiality. To address this, the questionnaire was modified to emphasize the anonymous nature of the data collection and to assure students that their responses would not be shared with teachers or school administrators. Finally, minor adjustments were made to the wording of some items to ensure cultural relevance and avoid any potential misinterpretations.

In addition to the name generator, four follow-up items were asked for each nominated friend: (a) frequency of academic discussion, (b) frequency of non-academic interaction, (c) perceived academic support, and (d) perceived emotional support. Each item was rated on a 5-point Likert scale (1 = never to 5 = very often/very much). Summed across all listed friends, these ratings yielded continuous indicators of academic interaction and emotional support that could be analyzed alongside structural metrics. These items were included to capture the relational quality of social capital, such as trust and support, complementing the structural measures like network size, density, and centrality (Coleman, 1988). For example, network centrality reflects access to resources and information, while density indicates the strength of bonding ties within peer groups (Lin, 1999). Because every student answered the same four Likert-type questions, the resulting item set (24 observed variables: 4 items × up to 6 friends, with missing values coded as 0) was suitable for confirmatory factor analysis (CFA).

The modified SNI was reviewed by experts in social network analysis for content validity, and the pilot test confirmed that the changes improved both comprehension and the quality of responses. These adaptations ensured that the instrument captured relevant social dynamics while maintaining high reliability (Cronbach's α > 0.85) and internal consistency, consistent with prior studies. The CFA treated the 24 indicators as reflective of a single latent factor labeled “Perceived Network Support.” This factor aligns with the relational dimension of social capital, emphasizing the trust and mutual support embedded in students' networks. The CFA results supported the unidimensional structure of the SNI, with acceptable model fit indices: χ^2^/*df* = 2.31, CFI = 0.95, TLI = 0.93, RMSEA = 0.06 (90% CI = 0.04–0.08), SRMR = 0.05.

#### 3.2.2 Academic self-efficacy

The Academic Self-Efficacy Scale (ASES) developed by Pintrich and De Groot (1990). The ASES is a widely used measure that assesses students' confidence in their ability to perform academic tasks. This scale includes items rated on a Likert scale from 1 (strongly disagree) to 5 (strongly agree). Reliability of the ASES has been well-documented, with Cronbach's alpha values typically above 0.80 (Pintrich and De Groot, 1990). For this study, the ASES was translated into Chinese and back-translated to ensure linguistic accuracy. A pilot test with a subset of students confirmed the scale's clarity and relevance. Given the translation and adaptation of the ASES, CFA was also employed to assess its construct validity within the Chinese sample. The model fit indices indicated a good fit to the data, confirming the scale's structural validity: χ^2^/*df* = 2.85, CFI = 0.92, TLI = 0.90, RMSEA = 0.07 (90% CI: 0.05, 0.09), SRMR = 0.06.

#### 3.2.3 Life satisfaction

The Satisfaction with Life Scale (SWLS) developed by Diener et al. (1985). The SWLS is a brief, five-item instrument designed to measure global cognitive judgments of one's life satisfaction. Participants rate each item on a 7-point Likert scale ranging from 1 (strongly disagree) to 7 (strongly agree). The SWLS has been extensively validated, with high internal consistency (Cronbach's alpha typically around 0.87) and good test-retest reliability (Diener et al., 1985). The scale was translated into Chinese, with back-translation procedures ensuring accuracy. A pilot test confirmed its applicability and comprehension among the target population. Similarly, CFA was performed on the translated SWLS to evaluate its construct validity. The results demonstrated acceptable model fit, supporting the use of the SWLS in this population: χ^2^/*df* = 3.12, CFI = 0.91, TLI = 0.88, RMSEA = 0.08 (90% CI: 0.06, 0.10), SRMR = 0.07.

#### 3.2.4 Interview

A semi-structured interview guide was designed for this study to examine student social networks, perceived academic support from friends, and how these networks influenced academic goals and wellbeing. The guide featured open-ended questions with prompts to encourage detailed responses and capture students' experiences. A pilot test with two students from the initial SNA sample was conducted to evaluate the clarity and flow of the questions and their ability to elicit meaningful data. Minor adjustments were made to the wording and order of questions based on the feedback, improving the interview process for participants.

The interview guide was developed iteratively, informed by existing literature on social capital, academic self-efficacy, and student wellbeing in rural settings. The pilot test ensured the questions were appropriate for the target population and produced relevant insights. To ensure the trustworthiness of the data, member checking was employed. After each interview, participants reviewed a summary of their key points and provided feedback on its accuracy. This process allowed for clarification or amendments, enhancing the credibility of the findings.

### 3.3 Procedure

This mixed-methods study employed a two-phase approach, commencing with a quantitative phase using social network analysis (SNA) followed by a qualitative phase involving narrative inquiry. Initial contact was established with school principals from two rural high schools in Sichuan and Heilongjiang provinces, China. Following the acquisition of informed consent from the principals, teachers facilitated participant recruitment. Information sheets detailing the study's purpose, procedures, and participant rights were distributed to students in grades 10–12. Written consent was obtained from students aged 18 or older, and parental consent was secured for those under 18.

During a designated class period, students completed a self-administered questionnaire. Researchers were available to address queries and ensure proper completion. Completed questionnaires were collected anonymously. Data completeness was verified, and friendship nominations were coded using Gephi and UCINET SNA software. Key network metrics—network size (number of unique nominations), density (ratio of actual to possible connections within the ego network), and degree centrality (number of direct ties, computed using UCINET algorithms)—were calculated. Gephi and UCINET were used for visualization and computation, with results cross-verified for reliability. The SNI instrument's reliability was confirmed through a pilot test (Cronbach's alpha > 0.85).

For the qualitative phase, 28 students were purposively selected from the initial SNA sample based on variation in network characteristics (size, density), academic self-efficacy scores, and gender balance to ensure diverse perspectives. These students were individually contacted and invited for semi-structured interviews (45–60 min) held in private school locations, with informed consent obtained prior to scheduling and audio-recording. The interview guide explored students' perceptions of their social networks, friendships, interaction frequency and nature, and the influence of these relationships on academic goals, study habits, and wellbeing.

All interviews were transcribed verbatim by a professional service. Researchers reviewed transcripts for accuracy, removing identifying information. An iterative coding process identified key themes related to social capital, academic self-efficacy, and life satisfaction, with multiple coding rounds ensuring comprehensive theme development (Saldaña, 2016). Data integration occurred during analysis, using SNA metrics and academic self-efficacy scores to group students for deeper qualitative data analysis. This triangulation facilitated cross-validation between quantitative network data and qualitative narratives, enriching the understanding of how network structures and student perceptions influenced academic self-efficacy and life satisfaction (Creswell and Plano Clark, 2017). The integrated analysis provided insight into the dual role of social networks—as sources of support and potential stressors—within the academic lives of students in rural China.

### 3.4 Data analysis

This mixed-methods study employed both quantitative and qualitative approaches for a comprehensive examination of the interplay between social networks, academic self-efficacy, and life satisfaction among rural Chinese high school students. Descriptive statistics summarized the sample's demographic characteristics (age, gender, grade level) and key variables (network size, density, academic self-efficacy, life satisfaction). Social network analysis (SNA) was conducted using UCINET (Borgatti et al., 2002) and Gephi (Bastian et al., 2009) to examine the structural properties of students' social networks, computing metrics such as density, centrality, and ego-network characteristics to identify patterns of social interaction and support. Inferential statistics, including independent samples *t*-tests and ANOVAs, examined differences in academic self-efficacy and life satisfaction across demographic and network characteristics. Multiple regression analysis in SPSS (Version 27) assessed the predictive power of social network variables on academic self-efficacy and life satisfaction, controlling for demographics. The reliability of the scales was confirmed using Cronbach's alpha, with values exceeding 0.80 indicating high internal consistency.

The qualitative data from semi-structured interviews were analyzed using thematic analysis, following Braun and Clarke's (2006) guidelines. Transcribed interviews were imported into NVivo (version 12) to facilitate coding. An initial coding scheme, based on research questions and literature review, included codes like “peer support,” “academic pressure,” and “family influence.” Open coding allowed for emergent codes (e.g., “shared struggles,” “fear of falling behind”). The coding process was iterative, with multiple researchers independently coding a subset of transcripts to ensure inter-coder reliability, resolving discrepancies through consensus. Constant comparison refined and merged codes into broader themes (e.g., “The Value of Supportive Friendships”). Axial coding explored relationships between themes. Member checking with participants enhanced the trustworthiness of qualitative findings (Lincoln and Guba, 1985).

## 4 Results

### 4.1 Quantitative phase

The quantitative phase of this study involved the analysis of data collected through the Social Network Index (SNI), the Academic Self-Efficacy Scale (ASES), and the Satisfaction with Life Scale (SWLS) from 454 rural high school students in China. The results provide insights into the social network structures, levels of academic self-efficacy, and life satisfaction among the participants.

#### 4.1.1 Descriptive statistics

The sample consisted of 454 rural high school students from China, with 39% male (*n* = 177) and 61% female (*n* = 277). The average age of the participants was 17.2 years (SD = 0.8), with a slight positive skewness (0.2) and negative kurtosis (−0.5), indicating a relatively normal age distribution.

Regarding the grade distribution, the sample was evenly spread across grades 10 (33%, *n* = 150), 11 (34%, *n* = 154), and 12 (33%, *n* = 150), ensuring a balanced representation of students at different stages of their high school education.

The descriptive statistics (Table 2) provide insights into participants' demographics, social network structures, academic self-efficacy, and life satisfaction. The average network size (number of close friends) was 4.3 (*SD* = 0.9), showing a near-normal distribution (*skewness* = 0.1, *kurtosis* = −0.6). Network density, the proportion of actual to possible connections, was 0.32 (*skewness* = 0.4, *kurtosis* = −0.7), indicating moderate connectivity. Degree centrality, the number of direct ties, averaged 3.7 (*SD* = 1.2) with *skewness* = 0.3 and *kurtosis* = −0.8.

**Table 2:** Descriptive statistics.

**Variable**	***N***	**%**	**Mean**	**SD**	**Skewness**	**Kurtosis**	**Cronbach's alpha**
**Gender**							
Male	177	39					
Female	277	61					
Age (years)	454	100	17.2	0.8	0.2	−0.5	
**Grade**							
Grade 10	150	33					
Grade 11	154	34					
Grade 12	150	33					
**Social network characteristics**							
Network size	454	100	4.3	0.9	0.1	−0.6	
Network density	454	100	0.32	0.09	0.4	−0.7	
Degree centrality	454	100	3.7	1.2	0.3	−0.8	
Academic self-efficacy	454	100	53.6	10.2	0.1	−0.4	0.89
Life satisfaction	454	100	24.8	6.3	−0.2	−0.5	0.87

The Academic Self-Efficacy Scale (ASES) had a mean score of 53.6 (*SD* = 10.2), with high internal consistency (Cronbach's α = 0.89) and a near-normal distribution (*skewness* = 0.1, *kurtosis* = −0.4), showing most students felt academically confident. The Satisfaction with Life Scale (SWLS) had a mean of 24.8 (*SD* = 6.3) with strong reliability (Cronbach's α = 0.87), slight negative *skewness* (−0.2), and moderate *kurtosis* (−0.5), suggesting generally moderate to high life satisfaction.

#### 4.1.2 Gender differences in academic self-efficacy and life satisfaction

Independent samples *t*-tests examined gender differences in academic self-efficacy and life satisfaction (Table 3). Females reported significantly higher self-efficacy (*M* = 54.8, *SD* = 9.8) than males (*M* = 51.2, *SD* = 10.6), *t*_(452)_ = 3.52, *p* < 0.001, with a moderate effect size (Cohen's *d* = 0.35), indicating gender's role in self-efficacy. No significant difference was found in life satisfaction between females (*M* = 24.6, *SD* = 6.2) and males (*M* = 25.1, *SD* = 6.5), *t*_(452)_ = −0.85, *p* = 0.396.This suggests that gender does not significantly impact life satisfaction in this sample.

**Table 3:** Gender differences in academic self-efficacy and life satisfaction.

**Variable**	**Gender**	***N***	**Mean**	**SD**	***t***	***p*-value**	**Cohen's *d***
Academic self-efficacy	Male	177	51.2	10.6	3.52	< 0.001	0.35
	Female	277	54.8	9.8			
Life satisfaction	Male	177	25.1	6.5	−0.85	0.396	–
	Female	277	24.6	6.2			

#### 4.1.3 Relationship between social network characteristics and academic self-efficacy

Pearson correlation analyses revealed significant positive correlations between network centrality and academic self-efficacy (*r* = 0.34, *p* < 0.001), and between network density and academic self-efficacy (*r* = 0.28, *p* < 0.001), as shown in Table 4. These findings suggest that students who hold more central positions in their social networks and those with denser networks tend to have higher academic self-efficacy. This highlights the importance of social network structure in influencing students' confidence in their academic abilities.

**Table 4:** Correlations between social network characteristics and academic self-efficacy.

**Variable**	**Network centrality**	**Network density**
Academic self-efficacy	0.34^**^	0.28^**^
Life satisfaction	0.20^**^	0.22^**^

^**^p < 0.001.

#### 4.1.4 Predictors of academic self-efficacy

A multiple regression analysis was conducted to identify the predictors of academic self-efficacy, incorporating network size, network density, degree centrality, gender, and grade level as predictors. The results, displayed in Table 5, indicate that the overall model was significant, *F*_(5, 448)_ = 23.45, *p* < 0.001, and explained 24.7% of the variance in academic self-efficacy (*R*^2^ = 0.247). Degree centrality (β = 0.31, *p* < 0.001) and network density (β = 0.27, *p* < 0.001) emerged as significant predictors, indicating that students with higher centrality and denser networks exhibit greater academic self-efficacy. Gender also emerged as a significant predictor (β = 0.18, *p* < 0.001), with female students reporting higher self-efficacy. Network size and grade level did not significantly predict academic self-efficacy (*p* > 0.05).

**Table 5:** Multiple regression analysis for predictors of academic self-efficacy.

**Predictor**	***B***	**SE**	**β**	***t***	***p*-value**
Network size	0.45	0.34	0.08	1.32	0.187
Network density	6.12	1.15	0.27	5.32	< 0.001
Degree centrality	4.87	0.76	0.31	6.41	< 0.001
Gender (female)	3.21	0.84	0.18	3.82	< 0.001
Grade level	0.15	0.41	0.02	0.37	0.711

R^2^ = 0.247, F_(5, 448)_ = 23.45, *p* < 0.001.

#### 4.1.5 Life satisfaction and social network characteristics

Pearson correlation analyses also showed a significant positive correlation between life satisfaction and network size (*r* = 0.22, *p* < 0.001), indicating that students with larger social networks reported higher life satisfaction. Additionally, network centrality was positively correlated with life satisfaction (*r* = 0.20, *p* < 0.001). These correlations, presented in Table 4, suggest that students who are more central in their social networks and those with more extensive networks tend to experience higher life satisfaction.

#### 4.1.6 Network characteristics across grades

**Figure 1:**
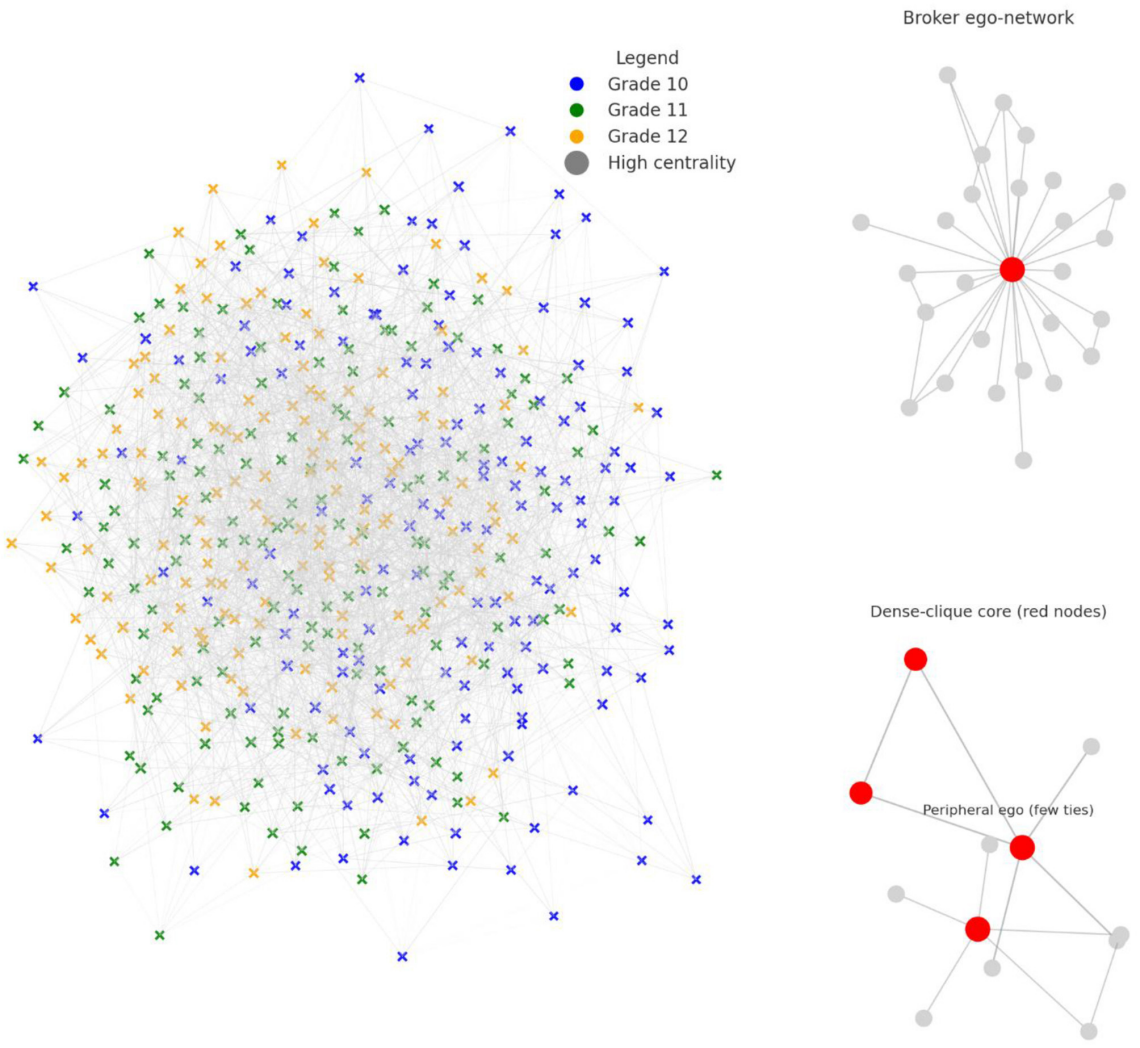
Friendship network for 454 rural Chinese high school students. Nodes are colored by grade (blue = 10, green = 11, orange = 12) and sized by degree centrality. Edge opacity scales with tie strength (stronger ties are darker). Insets show: the ego network of a high centrality “broker” (top right); the core of a dense clique, with nodes colored by grade (bottom right, upper portion); and an ego in a peripheral position with few ties (bottom right, lower portion).

ANOVA was conducted to assess differences in network characteristics across grades. The results (Table 6) showed significant variation in network density, *F*_(2, 451)_ = 6.78, *p* = 0.001. Grade 12 students had the highest network density (M = 0.35, SD = 0.07), compared to grade 10 (M = 0.29, SD = 0.09) and grade 11 (M = 0.31, SD = 0.08). *Post-hoc* Tukey HSD tests confirmed that the differences between grade 12 and both grades 10 and 11 were significant (*p* < 0.05). These results suggest that as students advance in high school, their social networks become denser, reflecting stronger peer connections in the final year. Figure 1 illustrates the friendship network structure, with nodes colored by grade and sized by degree centrality, highlighting the higher network density in Grade 12 (see Table 6). This visualization also informed subsequent qualitative themes by revealing patterns of social integration and isolation.

**Table 6:** ANOVA results for network density across grades.

**Grade**	***N***	**Mean**	**SD**	***F***	***p*-value**
Grade 10	150	0.29	0.09	6.78	0.001
Grade 11	154	0.31	0.08		
Grade 12	150	0.35	0.07		

*Post-hoc* Tukey HSD: Grade 12 > Grade 10, *p* < 0.05; Grade 12 > Grade 11, *p* < 0.05.

In summary, these findings highlight the role of social networks in students' academic self-efficacy and life satisfaction. Female students reported higher self-efficacy than males, and social network centrality and density were positively correlated with both academic confidence and life satisfaction. The variation in network density across grades emphasizes the increasing importance of peer relationships in later high school years.

### 4.2 Qualitative findings: narrative inquiry

The semi-structured interview questions were designed to explore students' perceptions of their friendships, the academic and emotional support they received, and the pressures they faced within their social circles. Specific questions included: “How do your friends help you with schoolwork?” “Do you ever feel pressure from your friends to perform better academically?” and “How do your relationships with family or relatives influence your school life?” These questions aligned with the study's main themes of supportive friendships, competitive pressures, and broader social networks.

The coding process followed a thematic analysis approach (Braun and Clarke, 2006). Initial open coding identified recurring ideas such as “peer encouragement,” “academic comparison,” and “family guidance.” These codes were then refined and grouped into broader themes through axial coding, resulting in three key themes that captured the nuanced ways social networks shaped students' experiences. The themes were further triangulated with SNA data to examine how network characteristics—such as centrality, density, and size—related to students' narratives.

#### 4.2.1 Theme 1: centrality and perceptions of supportive friendships

Students with high network centrality (many direct connections) frequently described their friendships as a key source of academic and emotional support. These students, often central in their social networks, reported feeling empowered by their numerous ties. For example, a Grade 11 student with high centrality and high academic self-efficacy said, “I have a lot of friends who are always willing to help. If I don't understand something, I can ask different people and get different perspectives.” This aligns with the quantitative finding that higher centrality correlates with greater academic self-efficacy (*r* = 0.34, *p* < 0.001).

In contrast, students with low centrality expressed feelings of isolation, which impacted their confidence. A Grade 10 student with low centrality and lower self-efficacy noted, “I don't have many close friends here, so when I struggle, I feel like I'm on my own. It makes me doubt whether I can keep up.” This triangulation shows how centrality not only provides access to resources but also shapes students' perceptions of their academic abilities.

#### 4.2.2 Theme 2: network density and experiences of competition

Students in denser networks—where friends were more interconnected—reported a complex relationship with competition. While some found the close-knit nature motivating, others felt overwhelmed by constant comparison. A Grade 12 student in a high-density network with moderate self-efficacy explained, “Everyone in my group knows each other's grades, so there's always this pressure to do better. It pushes me, but sometimes it's too much.” This reflects the quantitative finding that network density is positively correlated with academic self-efficacy (*r* = 0.28, *p* < 0.001), but also adds nuance by showing density can increase stress.

Students in less dense networks felt less competitive pressure. A Grade 11 student with a low-density network and high life satisfaction remarked, “My friends aren't all connected, so there's less talk about who's doing better. It's more relaxed, and I can focus on my own progress.” This suggests that while denser networks may boost academic confidence for some, they can also heighten anxiety for others, a tension not fully captured in the quantitative data.

#### 4.2.3 Theme 3: network size and the role of broader social support

Beyond in-school friendships, students with larger social networks—including family and relatives—described a broader support system that enhanced their wellbeing. A Grade 10 student with a large network and high life satisfaction shared, “I have a big family, and they're always there for me. Even if school gets tough, I know I have people to lean on.” This aligns with the quantitative correlation between network size and life satisfaction (*r* = 0.22, *p* < 0.001).

Students with smaller networks often relied heavily on a few key relationships, which could be both a strength and a vulnerability. A Grade 12 student with a small network but high academic self-efficacy said, “I don't have many friends, but my parents are very involved. They push me to do well, and that's enough for me.” This highlights how smaller, intimate networks can still provide critical support, especially with strong family involvement.

Overall, triangulating SNA and interview data reveals that network characteristics shape students' experiences in distinct ways. High centrality amplifies the benefits of supportive friendships, while dense networks can foster both motivation and stress. Larger networks, including family ties, contribute to wellbeing, offering a buffer against academic pressures. These refined themes provide a nuanced understanding of how social networks influence rural students, extending beyond generic ideas of support and competition.

## 5 Discussion

The present study explored the intricate role of social networks in shaping academic self-efficacy and life satisfaction among rural Chinese high school students. Our mixed-methods approach, integrating quantitative social network analysis with qualitative narrative inquiry, provided a comprehensive understanding of how peer relationships, family support, and social capital influence students' academic and emotional wellbeing. Drawing on social capital theory, we aimed to elucidate how network structures and relational dynamics contribute to these outcomes, and our findings offer nuanced insights into this process.

### 5.1 Social network structures and academic self-efficacy

Our quantitative findings revealed a significant positive association between social network centrality and density, and academic self-efficacy among rural Chinese high school students. This directly supports social capital theory, demonstrating higher academic confidence among students with greater embeddedness in strong, interconnected social networks (Dika and Singh, 2002; Putnam, 2000). Specifically, the positive correlation between network centrality, a manifestation of bridging social capital, indicates that access to diverse resources and information bolsters students' belief in their academic capabilities (Lin, 1999). Similarly, the importance of network density, reflecting bonding social capital, suggests that cohesive peer groups provide a supportive environment enhancing self-efficacy (Ahn, 2012). Qualitative data corroborate these findings, with students describing how supportive friendships, indicative of strong bonding capital, facilitated collaborative learning and provided crucial academic and emotional encouragement, as exemplified by a Grade 11 student with high centrality stating the benefit of diverse perspectives accessible through numerous connections. This aligns with Bandura's (1994) social cognitive theory, where self-efficacy is nurtured within supportive social contexts. Furthermore, qualitative insights highlighted the role of family support as linking social capital, connecting students to essential resources and long-term guidance, particularly vital in resource-constrained rural environments (Woolcock, 1998). The potential of bridging social capital, through connections with diverse peers, to enhance self-efficacy by exposing students to varied problem-solving approaches (Kyne and Aldrich, 2020; Lin, 1999) was also supported by student narratives.

### 5.2 Gender differences in academic self-efficacy

A notable finding was that female students reported significantly higher academic self-efficacy than their male counterparts. This result, while diverging from some earlier research, resonates with more recent studies indicating evolving gender dynamics in education (Llorca et al., 2017; Ma et al., 2016). In the Chinese context, this might reflect shifting societal expectations and educational practices that increasingly encourage female academic engagement (Fan and Chen, 2001; Hill, 2015). Our finding aligns with social role theory (Eagly and Wood, 2016), suggesting that in rural China, societal norms may encourage girls to prioritize academic effort and seek social support more readily than boys, potentially fostering higher levels of self-efficacy. The emphasis on diligence and relational interdependence in female socialization within this cultural context (Mishra, 2020; Murray et al., 2020) might also contribute to enhanced academic self-regulation and a greater propensity to engage in collaborative learning and help-seeking behaviors (Topping, 2005; Van der Zanden et al., 2018), further reinforcing their self-efficacy. However, it is important to consider that gender differences in self-efficacy can be domain-specific (Niehaus and Adelson, 2014), and future research could explore these nuances within our study population.

### 5.3 The dual nature of social networks: support and pressure

Our qualitative findings corroborated the dual nature of social networks in students' academic lives, acting as both sources of support and pressure, consistent with existing literature (Fox and Moreland, 2015; Nández and Borrego, 2013). Supportive peer relationships fostered learning and provided crucial emotional reinforcement, thereby enhancing both academic self-efficacy and life satisfaction (Eccles and Roeser, 2015; Wang et al., 2018). Students' descriptions of collaborative learning and mutual encouragement underscored the benefits of bonding social capital in promoting belonging and motivation (Ahn, 2012; Murray et al., 2020; Van Herpen et al., 2020). The qualitative data also highlighted the vital role of peer emotional support in mitigating academic stress and fostering resilience (Booker, 2007; Lee et al., 2023). Conversely, the pressure to meet peer achievements and the presence of unspoken competition within social circles frequently led to heightened anxiety and reduced self-confidence (Feinstein et al., 2013; Vogel et al., 2015), aligning with Festinger's (1954) social comparison theory. The influence of network structure was also evident, with students in denser networks often reporting greater competitive pressures (Felisoni and Godoi, 2018; Giunchiglia et al., 2018), suggesting that while density, representing strong bonding capital, can foster support, it can also amplify the negative effects of social comparison within close-knit groups. This was illustrated by a Grade 12 student in a high-density network who admitted, “Everyone in my group knows each other's grades, so there's always this pressure to do better. It pushes me, but sometimes it's too much.”

### 5.4 Family support as a distinct and critical influence

This study strongly emphasizes the crucial and distinct role of family support in shaping the academic self-efficacy and life satisfaction of rural high school students. Our qualitative findings revealed that family relationships provide essential emotional support and long-term guidance, which differs from the more immediate, task-focused support often offered by peers (Harris and Robinson, 2016; Roksa and Kinsley, 2019). The encouragement received from parents and extended family members was consistently linked to students reporting greater academic confidence and resilience, reinforcing the established importance of family involvement in fostering academic self-efficacy and motivation (Fan and Williams, 2010; Llorca et al., 2017; Mishra, 2020). In the context of resource-limited rural areas, family support often serves as a primary source of emotional and educational stability, effectively compensating for potential institutional deficits, thus acting as a vital form of linking social capital at the familial level (Baquedano-López et al., 2013; Boonk et al., 2018; Woolcock, 1998). Furthermore, family support appeared particularly vital for students who lacked strong in-school networks, playing a crucial role in maintaining their engagement and overall wellbeing, which aligns with research on the contributions of extended family (Galindo and Sheldon, 2012; Gueldner et al., 2020). As a Grade 12 student with a small network noted, “I don't have many friends, but my parents are very involved. They push me to do well, and that's enough for me,” highlighting the significance of family involvement for some students.

### 5.5 Educational inequities and social capital

The findings of this study highlight the significant role of social capital, manifested through social networks, in shaping educational outcomes within rural Chinese high schools, where socio-economic disparities are prevalent (Gorski, 2017; Shifrer et al., 2013). Our results directly illustrate how disparities in social capital are linked to educational inequities: students with smaller or weaker social networks, often from lower socio-economic backgrounds, exhibited lower levels of academic self-efficacy and life satisfaction. This aligns with the understanding that limited social capital can exacerbate existing educational inequities (Coleman, 1988; Putnam, 2000; Roscigno and Ainsworth-Darnell, 1999). For instance, qualitative data revealed that students with fewer close peer connections expressed isolation and a lack of confidence, supported by quantitative findings indicating positive correlations between network size and centrality with self-efficacy (*r* = 0.34, p < 0.001) and life satisfaction (*r* = 0.22, p < 0.001). These findings underscore the critical importance of fostering social capital in rural China, where underdeveloped infrastructure and scarce resources can deepen socio-economic gaps (Mishra et al., 2023; Roscigno et al., 2006). Qualitative interviews further emphasized the compensatory role of family support, a crucial form of linking social capital at the familial level, for students with weak peer networks, as exemplified by a Grade 12 student stating parental encouragement helps them stay on track despite having few friends. This highlights how strong family ties can buffer the negative impacts of limited peer social capital, particularly for disadvantaged students (Baquedano-López et al., 2013; Becker and Luthar, 2002; Woolcock, 1998). Our study's findings strongly support the notion that interventions aimed at enhancing social capital through initiatives like mentorship, community engagement, and parental involvement can be crucial in mitigating educational disadvantages and promoting equity (Bernardi and Ballarino, 2016).

## 6 Implications

The findings of this study have important implications for educational practice and policy. First, recognizing the dual nature of social networks is essential for educators aiming to promote positive peer interactions while minimizing competitive pressures. Implementing structured group activities that emphasize collaboration and shared goals can foster academic self-efficacy and life satisfaction among students. These activities create a supportive learning environment where teamwork is prioritized. Educators must also be alert to signs of unhealthy competition and provide resources such as counseling services and stress management workshops to help students cope with these pressures.

Second, strengthening family involvement in education is especially important in rural areas where schools often lack adequate resources. Schools can promote parent-teacher partnerships and create opportunities for parental involvement to support students' academic motivation and resilience. For instance, organizing workshops that teach parents how to support their children's learning at home can help reinforce these connections. Additionally, schools can partner with community organizations to enhance social capital and offer students broader support networks. Collaborating with local stakeholders can provide mentorship opportunities and after-school programs that enrich students' educational experiences.

Third, addressing educational inequities requires systemic efforts to expand social capital for disadvantaged students. Policymakers should ensure equitable resource allocation so that schools serving low-income and minority students receive the necessary funding to offer high-quality education and opportunities for social capital building. Programs such as mentorship initiatives, extracurricular activities, and peer-support groups can connect students with mentors and role models who provide both academic and personal guidance. Additionally, increasing awareness among educators and stakeholders about the value of inclusive school environments can encourage the adoption of practices that promote social integration and academic success for all students.

## 7 Limitations and future research

Despite the valuable insights gained, this study presents several limitations that offer promising avenues for future research. Firstly, the generalizability of our findings may be limited by the sample's geographic restriction to two rural high schools within China. Future research should aim to include a more diverse sample, encompassing a wider range of rural and urban schools across different regions, to enhance the broader applicability of the results. Secondly, the cross-sectional design of this study prevents us from establishing causal inferences between social network characteristics and academic self-efficacy. Longitudinal studies are warranted to provide deeper insights into the dynamic interplay between these factors, allowing researchers to track changes in students' social networks over time and assess their subsequent impact on academic outcomes.

Thirdly, while our Social Network Analysis effectively captured structural aspects of social capital, such as network centrality and density, it did not fully account for normative elements like trust and reciprocity. Although these aspects were explored through the qualitative phase, future research could benefit from integrating quantitative measures of relational quality to provide a more comprehensive assessment of social capital. Fourthly, the reliance on self-reported data may have introduced social desirability bias. To enhance the validity of future findings, researchers could incorporate objective measures of academic performance, as well as observational data on social interactions, to complement self-report measures.

Fifthly, while the qualitative phase yielded valuable in-depth insights, the relatively small sample size of 28 students may have limited the depth and breadth of the analysis. Future studies could consider expanding the qualitative component to explore additional themes and provide a more nuanced understanding of the multifaceted factors influencing academic self-efficacy and life satisfaction in this context. Finally, future research should consider examining the intersectionality of various factors, including socio-economic status, cultural background, and geographic location, to develop a more comprehensive understanding of the complex influences on academic outcomes among rural Chinese high school students. Furthermore, investigating the evolving role of digital social networks in this population, particularly given the increasing prevalence of online interactions, could illuminate contemporary dynamics shaping students' academic and emotional wellbeing.

## 8 Conclusion

In conclusion, this study underscores the complex role of social networks in shaping academic self-efficacy and life satisfaction among rural high school students in China. The findings reveal that while social networks offer essential support, they can also introduce pressures that affect students in varied ways. Understanding these multifaceted relationships is key for educators, policymakers, and stakeholders seeking to improve educational outcomes and promote equity. By fostering strong peer and family support systems and addressing the structural inequalities that hinder social capital, educational environments can be created that empower all students to succeed. Targeted interventions that strengthen social capital and provide both emotional and academic support can help reduce the impact of socio-economic disadvantages. Ultimately, an integrated approach that considers social, emotional, and structural factors is crucial for enhancing academic self-efficacy, life satisfaction, and overall student wellbeing.

## Data Availability

The data analyzed in this study is subject to the following licenses/restrictions: The datasets generated and/or analyzed during the current study are not publicly available due to privacy and ethical restrictions but are available from the corresponding author on reasonable request. Requests to access these datasets should be directed to Ping Zhu, zhuping1105@126.com.
